# Incidence and Outcomes of CNS Tumors in Chinese Children: Comparative Analysis With the Surveillance, Epidemiology, and End Results Program

**DOI:** 10.1200/JGO.19.00378

**Published:** 2020-05-11

**Authors:** Anthony P. Y. Liu, Qi Liu, Matthew M. K. Shing, Dennis T. L. Ku, Eric Fu, Chung-Wing Luk, Siu-Cheung Ling, Kevin K. F. Cheng, Dora L. W. Kwong, Wilson W. S. Ho, Ho-Keung Ng, Amar Gajjar, Yutaka Yasui, Godfrey C. F. Chan, Gregory T. Armstrong

**Affiliations:** ^1^ Department of Oncology, St Jude Children’s Research Hospital, Memphis, TN; ^2^ Department of Pediatrics and Adolescent Medicine, Li Ka Shing Faculty of Medicine, The University of Hong Kong, Hong Kong SAR, China; ^3^ Department of Public Health Sciences, University of Alberta, Edmonton, Alberta, Canada; ^4^ Department of Pediatrics and Adolescent Medicine, Hong Kong Children’s Hospital, Hong Kong SAR, China; ^5^ Department of Pediatrics and Adolescent Medicine, Princess Margaret Hospital, Hong Kong SAR, China; ^6^ Division of Neurosurgery, Department of Surgery, Queen Mary Hospital, Hong Kong SAR, China; ^7^ Division of Neurosurgery, Department of Surgery, Hong Kong Children’s Hospital, Hong Kong SAR, China; ^8^ Department of Clinical Oncology, Queen Mary Hospital, The University of Hong Kong, Hong Kong SAR, China; ^9^ Department of Anatomical and Cellular Pathology, Faculty of Medicine, The Chinese University of Hong Kong, Hong Kong SAR, China; ^10^ Department of Epidemiology and Cancer Control, St Jude Children’s Research Hospital, Memphis, TN

## Abstract

**PURPOSE:**

Despite being the most common pediatric solid tumors, incidence and outcome of CNS tumors in Chinese children have not been systematically reported. We addressed this knowledge gap by comparing the epidemiology of pediatric CNS tumors in Hong Kong and the United States.

**PATIENTS AND METHODS:**

Data between 1999 and 2016 from a population-based cancer registry in Hong Kong, China, on patients < 18 years old with CNS tumors (Hong Kong cohort) and from the US SEER Program (Asian/Pacific Islander and all ethnicities) were compared. Incidence and overall survival (OS) by histology were evaluated.

**RESULTS:**

During the study period, 526 children were newly diagnosed with CNS tumors in Hong Kong (crude incidence rate, 2.47 per 100,000; 95% CI, 2.26 to 2.69). Adjusted incidences were significantly lower in the Hong Kong (2.51; 95% CI, 2.30 to 2.74) than in the SEER (Asian/Pacific Islander: 3.26; 95% CI, 2.97 to 3.57; *P* < .001; all ethnicities: 4.10 per 100,000; 95% CI, 3.99 to 4.22; *P* < .001) cohorts. Incidences of germ cell tumors (0.57 *v* 0.24; *P* < .001) were significantly higher, but those of glial and neuronal tumors (0.94 *v* 2.61; *P* < .001), ependymomas (0.18 *v* 0.31; *P* = .005), and choroid plexus tumors (0.08 *v* 0.16; *P* = .045) were significantly lower in Hong Kong compared with SEER (all ethnicities) cohorts. Compared with the SEER (Asian/Pacific Islander) cohort, histology-specific incidences were similar except for a lower incidence of glial and neuronal tumors in Hong Kong (0.94 *v* 1.74; *P* < .001). Among cohorts, OS differed only for patients with glial and neuronal tumors (5-year OS: Hong Kong, 52.5%; SEER [Asian/Pacific Islander], 73.6%; SEER [all ethnicities], 79.9%; *P* < .001).

**CONCLUSION:**

We identified important ethnic differences in the epidemiology of CNS tumors in Chinese children. These results will inform the development of pediatric neuro-oncology services in China and aid further etiologic studies.

## INTRODUCTION

Cancer is a major cause of childhood mortality in developed regions of the world and an underestimated public health issue in children from low- and middle-income countries.^1^ Survival of pediatric patients with CNS tumors, the most common solid tumor in children, remains lower than survival for other cancer types.^2-4^ Epidemiologic studies are important to evaluate disease burden among regions and ethnic groups.^5-7^ In China, there has been increased focus on optimizing the care for children with cancer along with the country’s socioeconomic growth.^8^ The Chinese Ministry of Health has supported large-scale multi-institutional projects, and trials geared toward standardizing the care for children with leukemia are underway.^9-11^ However, additional efforts are required to systematically assess and address the needs of children with CNS tumors in China. The lack of population-based pediatric oncology registries, and thereby epidemiologic data specific to this population, further hinders the ability of health care providers, researchers, and policy makers to address the current challenges of providing optimal pediatric neuro-oncology services in China.^12-15^

Hong Kong is a Special Administrative Region of China that has a well-defined population because of historical, administrative, and geographic reasons. Hong Kong’s population is 7.17 million; 1.2 million residents are < 18 years old, and 94% are of Chinese ethnicity.^16^ Children with cancer have been managed at 5 government hospitals providing multidisciplinary specialist services. In 1993, these 5 pediatric oncology units formed the Hong Kong Pediatric Haematology/Oncology Study Group (HKPHOSG) to improve the standards of managing cancer or blood diseases in pediatric patients and to harmonize treatment strategies for specific conditions.^17^ The HKPHOSG also runs a population-wide database prospectively enrolling children with different oncologic conditions. Data are accrued by data managers at HKPHOSG and cross-checked with the Hong Kong Cancer Registry.

To address the lack of epidemiologic data on CNS tumors in Chinese children, we used population-based patient data from Hong Kong to determine the incidence and outcome of children with common CNS tumors during an 18-year period and compare them with those of patients of Asian descent and all ethnicities in the United States by using the SEER registry.

## PATIENTS AND METHODS

### Hong Kong Study Cohort

From the HKPHOSG database, information was extracted on all residents in Hong Kong < 18 years of age diagnosed with a primary CNS tumor between January 1999 and December 2016 (Hong Kong cohort). Data from nonresident patients (n = 47) were excluded. Information on demographics, clinical features, histology, tumor grading (when available), treatment received (surgery and extent, irradiation and extent, chemotherapy), and survival was included. Histologic diagnosis and grading were based on the report of pathologists at individual institutions, according to the WHO International Classification of Diseases for Oncology, third edition (ICD-O-3).^18,19^ Data from patients not undergoing biopsy or resection also were included, with diagnosis made according to neuroimaging findings. Additional demographic data for the overall Hong Kong population under the coverage of HKPHOSG were extracted and interpolated from the Hong Kong Government census reports from 1996, 2001, 2006, 2011, and 2016.^16^ The study was approved by the institutional review board of the University of Hong Kong, and need for informed consent was waived.

### SEER Cohorts

The SEER database was queried to identify patients < 18 years of age diagnosed with primary CNS tumors during the same period (1999-2016).^20^ Specifically, data on patients with tumor sites “brain and other nervous system” (ICD-O-3 site codes: C710-719, 700-709, 720-729), “pituitary gland” (C75.1), “craniopharyngeal duct” (C75.2), and “pineal gland” (C75.3) were included. Patients of all races/ethnicities and those limited to Asians/Pacific Islanders formed the SEER (all ethnicities) cohort and the SEER (Asian/Pacific Islander) cohort, respectively. Demographic features, histology, tumor grading (when available), and survival data for all ethnicities combined and for Asians/Pacific Islanders by age, sex, and year were downloaded for the same period.^21^ Extent of resection could be inferred from the variable “RX Summ–Surg Prim Site” for patients with tumor sites in the “brain and other nervous system” and categorized as having received gross total resection (30, 40, 55), biopsy/subtotal resection (10, 20, 21, 22), no surgery (00), or data not available (90, 99).

### Histologic Groups

Individual histologic diagnoses were further categorized into 6 main histologic groups to facilitate comparison among study cohorts according to the ICD-O-3.^19^ These included (1) choroid plexus tumors (CPTs; ICD-O-3 histology code 9390); (2) craniopharyngiomas (ICD-O-3 histology codes 9350, 9351); (3) embryonal tumors (medulloblastoma, ICD-O-3 histology codes 9470-9472, 9474; other embryonal tumors, ICD-O-3 histology codes 8963, 9362, 9364, 9473, 9490, 9500-9503, 9508); (4) ependymal tumors (ICD-O-3 histology codes 9383, 9391-9394); (5) germ cell tumors (GCTs; germinoma, ICD-O-3 histology codes 9060, 9064, 9065; nongerminomatous germ cell tumors [NGGCT], ICD-O-3 histology codes 9070, 9071, 9085, 9100, 9101; teratoma, ICD-O-3 histology codes 9080, 9081, 9084); and (6) glial and neuronal tumors (ICD-O-3 histology codes 9380-9382, 9384, 9400, 9401, 9412, 9413, 9420, 9421, 9424, 9430, 9440-9442, 9444, 9450, 9451, 9492, 9493, 9505, 9506). Glial and neuronal tumors were further classified according to histologic grading (WHO grades I-II, III-IV, and not available [NA]). This group also included entities that were diagnosed clinicoradiographically, such as diffuse intrinsic pontine glioma and optic pathway glioma. In the Hong Kong cohort, all glial and neuronal tumors without histologic grading did not receive tissue diagnosis. In the SEER cohort, the unavailability of tumor grading included a combination of tumors not having tissue diagnosis and those having missing data.

### Statistical Analyses

Date of diagnosis was defined as the date of first resection/biopsy or the date of first neuroimaging in cases wherein tissue was not obtained. Follow-up started from the date of diagnosis, and patient data were censored at the time of loss of follow-up or December 31, 2016, whichever was earlier. Overall survival (OS) was calculated from the date of diagnosis to the date of death or last follow-up (censored) by using the Kaplan-Meier method. Survival curves were compared between cohorts stratified by histologic subgroups and by different clinical characteristics in the Hong Kong cohort, such as sex, age at diagnosis (0-4, 5-9, 10-14, 15-< 18 years), period of diagnosis (1999-2007, 2008-2016), WHO grading (I-II, III-IV), and histologic groups using the log-rank test. Incidence rates were estimated and compared among cohorts overall and stratified by histologic subgroups, with analysis adjusted for age, sex, and study period using Poisson regression models. A *P* value < .05 was considered significant. All statistical analyses were performed with SAS version 9.4 (SAS Institute, Cary, NC).

## RESULTS

### Demographic and Clinical Characteristics of Patients in the Hong Kong Cohort

**FIG 1:**
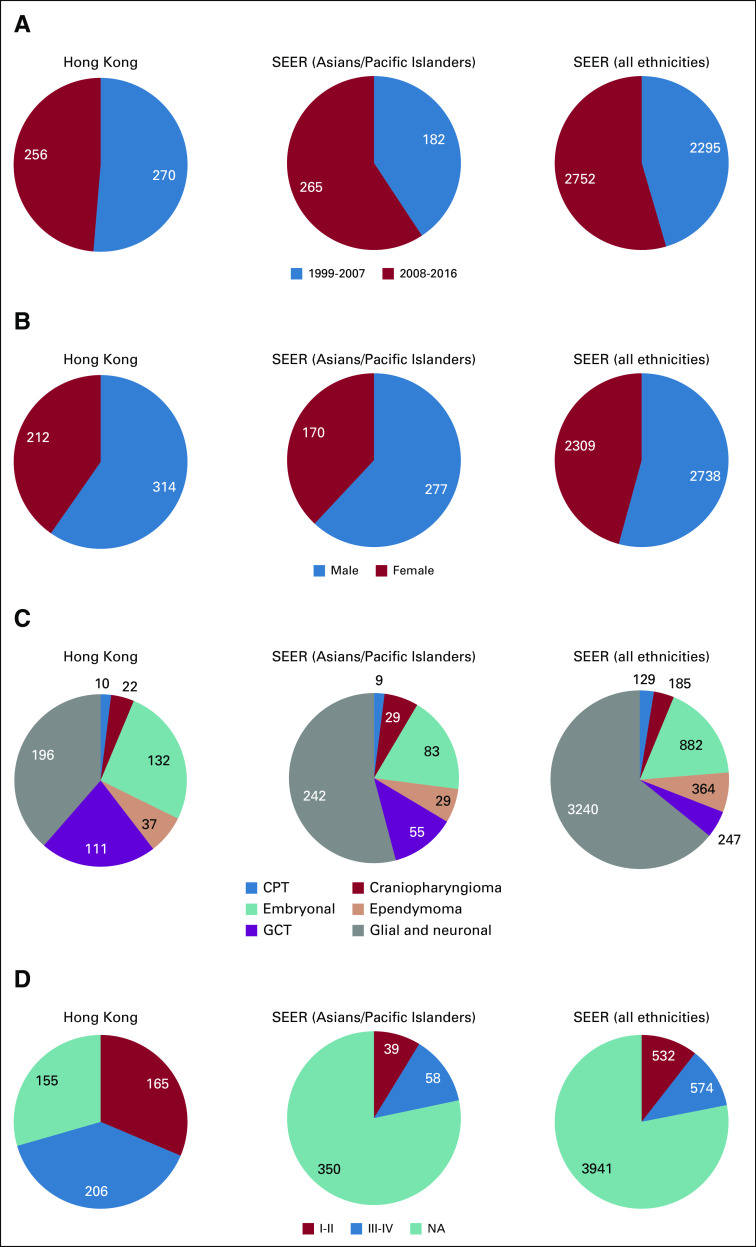
Distribution of patients by (A) period of diagnosis, (B) sex, (C) diagnostic categories, and (D) WHO grading in the Hong Kong, SEER (Asians/Pacific Islanders), and SEER (all ethnicities) cohorts. CPT, choroid plexus tumor; GCT, germ cell tumor; NA, not available.

During the study period, 526 patients < 18 years of age were newly diagnosed with CNS tumors in Hong Kong (1999-2007: n = 270 [51.3%]; 2008-2016: n = 256 [48.7%]). The crude incidence rate was 2.47 per 100,000 children (95% CI, 2.26 to 2.69; Table 1; Appendix Table A1, online only). The male-to-female ratio was 1.48, and the mean age of diagnosis was 8.8 years (interquartile range, 4.4-13.1 years; Fig 1). Of the patients, 511 (97%) were of Chinese ethnicity. Diagnosis was based on histology for 473 patients (including autopsy for 1 patient and cerebrospinal fluid cytology only for 1 patient) and on radiographic features and/or biochemical markers for 53 patients (brainstem glioma, n = 32; GCT, n = 13; optic pathway glioma, n = 3; and other, n = 5). For histologically diagnosed tumors and samples having assigned WHO grading, 165 and 206 patients had grade I-II and grade III-IV tumors, respectively.

**TABLE 1:**
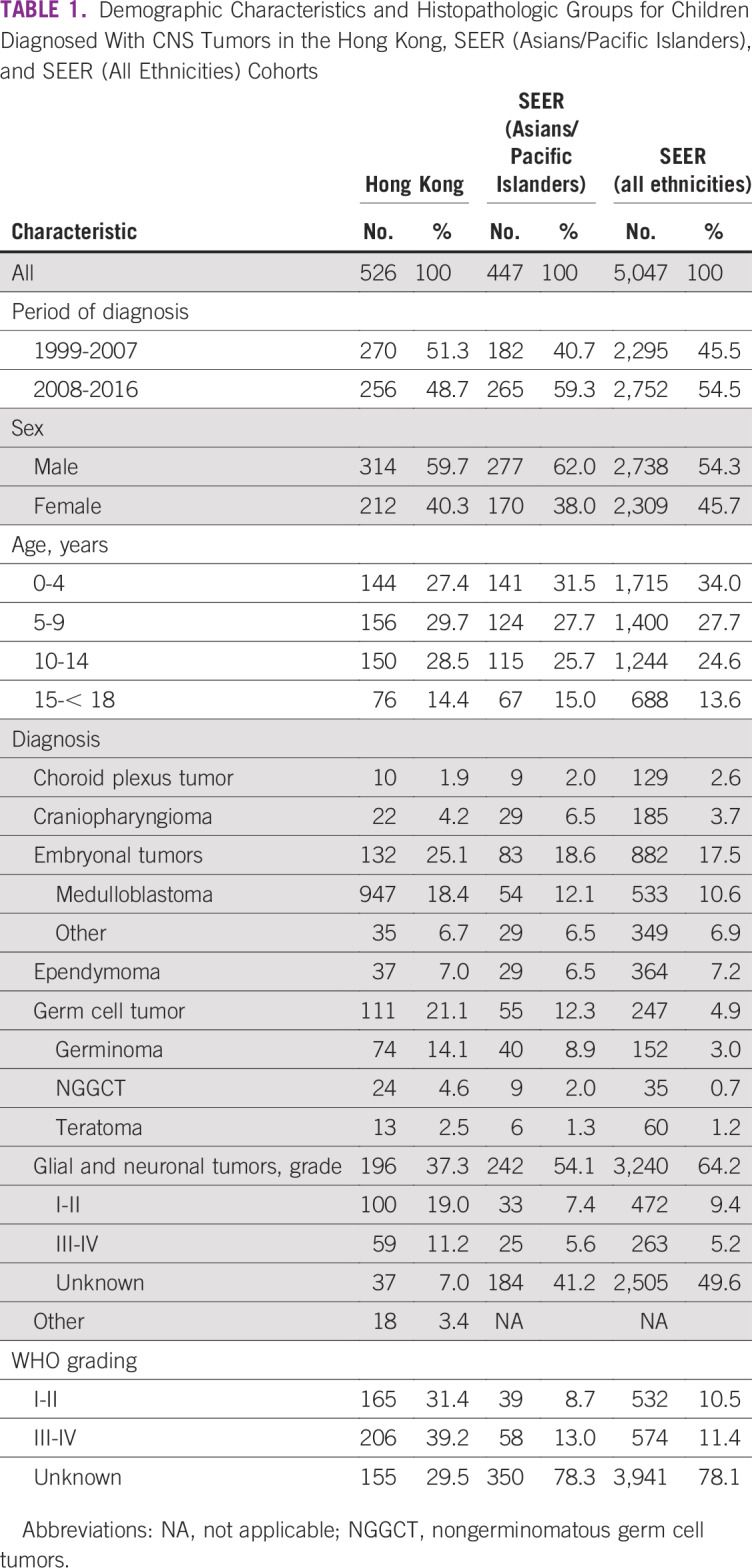
Demographic Characteristics and Histopathologic Groups for Children Diagnosed With CNS Tumors in the Hong Kong, SEER (Asians/Pacific Islanders), and SEER (All Ethnicities) Cohorts

### Overall Incidence Rates Among Study Cohorts

**FIG 2:**
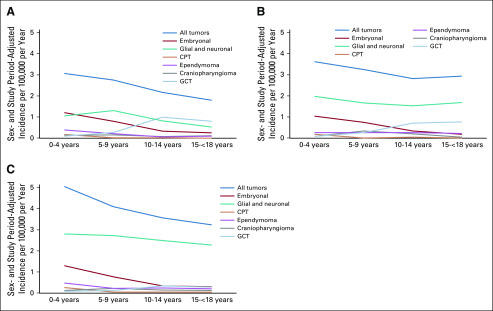
Sex- and study period–adjusted incidence of histologic groups by age in the (A) Hong Kong, (B) SEER (Asians/Pacific Islanders), and (C) SEER (all ethnicities) cohorts. CPT, choroid plexus tumor; GCT, germ cell tumor; y, years.

In the Hong Kong cohort, 508 patients were diagnosed with tumors belonging to the 6 predefined histologic groups compared with 447 in the SEER (Asian/Pacific Islander) cohort and 5,047 in the SEER (all ethnicities) cohort. Corresponding age-, sex-, and study period–adjusted incidence rates were 2.51 (95% CI, 2.30 to 2.74), 3.26 (95% CI, 2.97 to 3.57), and 4.10 (95% CI, 3.99 to 4.22), respectively (Table 2; Fig 2). Adjusted incidence rates were significantly lower in the Hong Kong cohort than in the SEER (Asian/Pacific Islander; rate ratio [RR], 0.77; 95% CI, 0.68 to 0.87; *P* < .001) and SEER (all ethnicities; RR, 0.61; 95% CI, 0.56 to 0.67; *P* < .001) cohorts. Incidence rates of all CNS tumors decreased with age in all 3 pediatric cohorts (Fig 2). Demographic features for each cohort are illustrated in Figure 1.

**TABLE 2:**
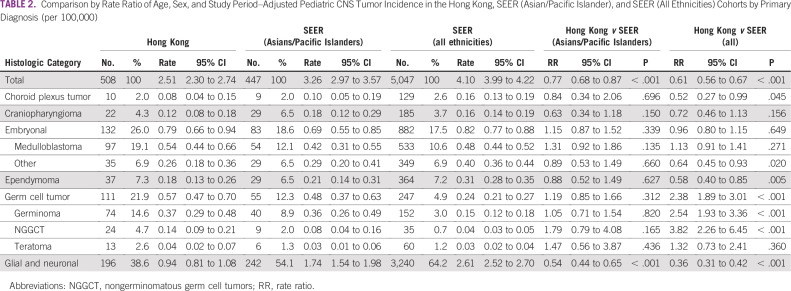
Comparison by Rate Ratio of Age, Sex, and Study Period–Adjusted Pediatric CNS Tumor Incidence in the Hong Kong, SEER (Asian/Pacific Islander), and SEER (All Ethnicities) Cohorts by Primary Diagnosis (per 100,000)

### Comparison of Incidence Rates by Histology

In the Hong Kong and SEER (Asian/Pacific Islander) cohorts, glial and neuronal tumors were the most common histologic group, followed by embryonal tumors and GCTs (Table 2; Fig 1). This was in contrast to the SEER (all ethnicities) cohort, for which ependymomas, and not GCTs, were the third most common histologic group. Comparison of adjusted incidence rates between the SEER (all ethnicities) and Hong Kong cohorts showed significantly higher rates of GCTs (RR, 2.38; 95% CI, 1.89 to 3.01; *P* < .001) but lower rates of glial and neuronal tumors (RR, 0.36; 95% CI, 0.31 to 0.42; *P* < .001), ependymomas (RR, 0.58; 95% CI, 0.40 to 0.85; *P* = .005), and CPTs (RR, 0.52; 95% CI, 0.27 to 0.99; *P* = .045) in the Hong Kong cohort. Adjusted incidence rates for both germinoma and NGCCTs were significantly higher in the Hong Kong cohort than in the SEER (all ethnicities) cohort (RR, 2.54 [95% CI, 1.93 to 3.36] and 3.82 [95% CI, 2.26 to 6.45], respectively; *P* < .001 for both). Histologic group-specific incidence rates were not significantly different between the SEER (Asian/Pacific Islander) and Hong Kong cohorts except for glial and neuronal tumors, for which the incidence rate was lower in the Hong Kong cohort (RR, 0.54; 95% CI, 0.44 to 0.65; *P* < .001) than the SEER cohort. Of note, adjusted incidence rates for GCTs were almost identical in the Hong Kong and SEER (Asian/Pacific Islander) cohorts.

### Treatment Characteristics and Outcomes of the Hong Kong Cohort

**FIG 3:**
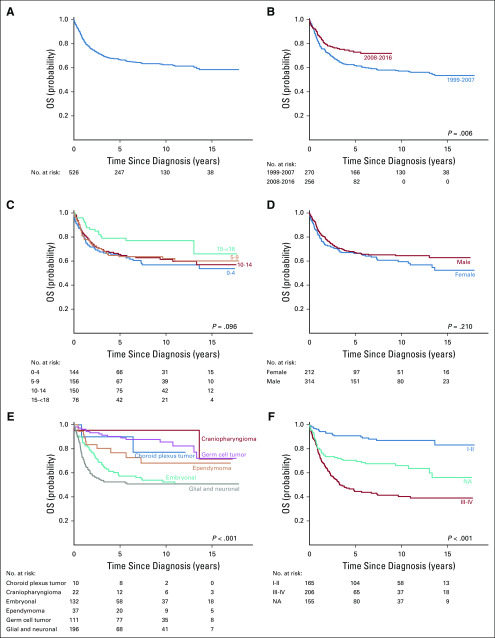
Five-year overall survival (OS) of patients in the Hong Kong cohort: (A) entire cohort and by (B) period of diagnosis, (C) age of diagnosis, (D) sex, (E) diagnostic categories, and (F) WHO grading. NA, not available.

Biopsy or resection was performed in 470 patients (89%); gross total resection was achieved in 258 patients (49%; Table 3), similar to rates in the SEER (all ethnicities) cohort (2,276 [48.8%] of 4,656; *P* = .844; Appendix Table A2, online only). Patients who did not undergo tumor-directed surgeries included mostly those with brainstem gliomas or GCTs. Radiotherapy was given to 314 patients (60%) and chemotherapy, to 328 patients (62%); treatment strategies varied by histologic diagnosis. The mean follow-up duration was 5.9 years. The 5-year OS for the entire Hong Kong cohort was 66.8% (95% CI, 62.3 to 70.8; Fig 3; Appendix Table A3, online only). Patients diagnosed more recently (2008-2016) had a significantly better OS (*P* = .006) than those diagnosed in the earlier period (1999-2007), as did those with low-grade tumors (WHO grade I-II; *P* < .001). Patients with embryonal or glial and neuronal tumors had inferior OS (*P* < .001). Period of diagnosis, tumor grade, and diagnostic categories remained significant predictors of outcome in multivariable analysis (Appendix Table A4, online only). Sex and age at diagnosis did not significantly affect survival.

**TABLE 3:**
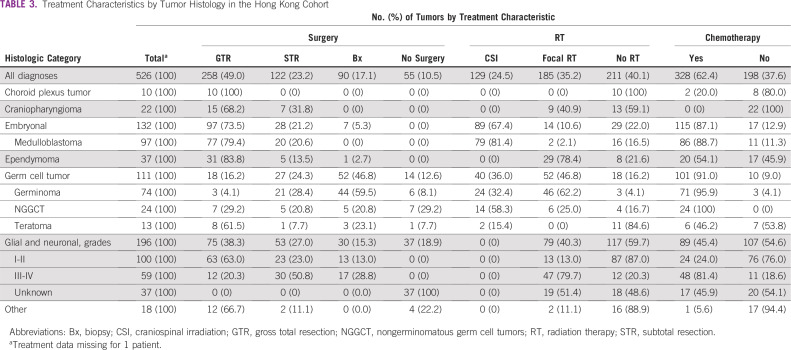
Treatment Characteristics by Tumor Histology in the Hong Kong Cohort

### Comparison of Survival Among the Hong Kong and SEER Cohorts

**FIG A1:**
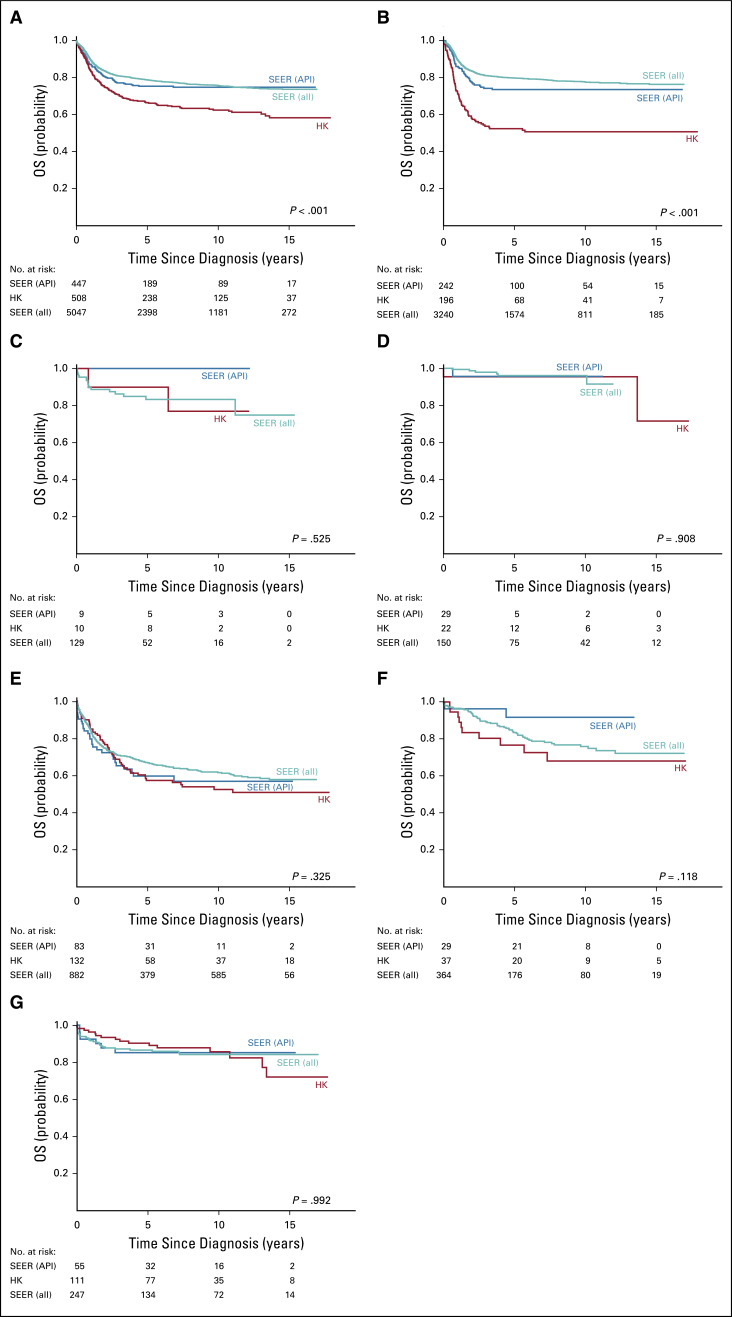
Comparison of overall survival (OS) for patients from the Hong Kong (HK), SEER (Asians/Pacific Islanders [API]), and SEER (all ethnicities [all]) cohorts: (A) all entities, (B) glial and neuronal tumors, (C) choroid plexus tumors, (D) craniopharyngioma, (E) embryonal tumors, (F) ependymoma, and (G) germ cell tumors.

**FIG A2:**
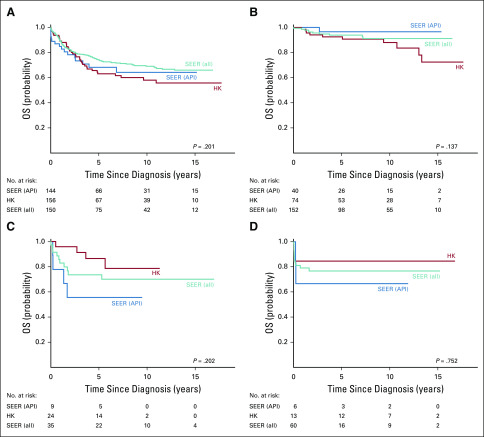
Comparison of overall survival (OS) of patients from the Hong Kong (HK), SEER (Asians/Pacific Islanders [API]), and SEER (all ethnicities [all]) cohorts by tumor subgroups: (A) medulloblastoma, (B) germinoma, (C) nongerminomatous germ cell tumors (NGGCTs), and (D) teratoma.

When patients from the entire cohort were compared, OS in patients from the Hong Kong cohort was lower than in those from the SEER (Asian/Pacific Islander) and SEER (all ethnicities) cohorts (*P* < .001; Appendix Table A5, online only; Appendix Fig A1, online only). By histology, similar discrepancies were seen in patients with glial and neuronal tumors (*P* < .001; Appendix Fig A1). No significant difference in OS was seen in patients with tumors of other histologies among the cohorts (Appendix Table A5; Appendix Fig A2, online only).

## DISCUSSION

An estimated 45,000 children are diagnosed with cancer in China every year, including 9,000-11,000 children with CNS tumors, assuming that 20%-25% of pediatric cancers are CNS in origin.^8,22^ Management of cancer in children in China is hampered by factors such as a largely privatized health care system, lack of health insurance, treatment abandonment, and limited coverage by population-based cancer registries.^8,12-15^ To our knowledge, our study is the first to directly compare the population-based incidence and OS for children with CNS tumors from China with those from patients in the United States.^4,22^ We observed a lower incidence of childhood CNS tumors in the Hong Kong cohort than in the SEER cohorts, even when comparing only Asian/Pacific Islanders in the registry. Existing epidemiologic data on pediatric cancer in China have generally been restricted to reports from major cities and on major cancer classes.^12,13,15^ For example, a study of cancer in children < 14 years of age from Shanghai between 2002 and 2005 revealed that 123 had CNS tumors, which accounted for 20.2% of all cancer cases and translated to an age-adjusted incidence of 2.40 per 100,000.^13^ This report, which was based on the International Classification of Childhood Cancer, separated patients with CNS GCTs, for which an adjusted incidence was 0.19 per 100,000. In a parallel report from Beijing that used International Classification of Childhood Cancer categories, the age-adjusted incidences of all CNS tumors and CNS GCTs in children < 14 years of age were 1.93 and 0.18 per 100,000, respectively, in 2000-2009.^15^ These findings approximate the incidence rate of CNS tumors identified in our study by using the population-based database in Hong Kong (2.48 per 100,000, including CNS GCTs), thereby suggesting a valid discrepancy in CNS tumor incidence among children in China and the United States.

Comparison cohorts in our study were built from the SEER registry with matched age range, period of diagnosis, and ICD-O-3 histologic coding. This allowed a head-to-head comparison of disease incidence and survival by histologic categories that are relevant to the latest consensus in CNS tumor classification.^18,23^ Previous studies have reported higher incidence rates of intracranial GCTs in Asians (11%-14%) than in the Western population (< 5%), although most of these reports were based on institutional experience and the proportion of cases rather than incidence rates.^24-28^ Our study strongly suggests a racial/ethnic predisposition for the development of CNS GCTs in Chinese children, with incidence rates more than twice those of the US population. The mechanism underlying such ethnic differences remains largely elusive, although the contribution from germline variants in *JMJD1C* to the development of intracranial GCT in Japanese patients has been reported.^29^ Existing evidence for ethnic differences in the incidence of ependymoma is limited. A review by the Brain Tumor Epidemiology Consortium that summarized reports on pediatric ependymoma and incidence rates in the Western population (Europe, Nordic countries, United Kingdom, United States) found rates ranging from 0.25 to 0.42 per 100,000 compared with 0.15 per 100,000 in Japan and 0.18 per 100,000 in our study.^26,30-35^ Our findings confirm these interstudy comparisons and suggest lower incidence rates of pediatric ependymoma in Chinese and Asians. Despite discrepancies in the availability of histologic grading for glial and neuronal tumors, our findings align with previous studies reporting lower incidence of such entities in Asians than in non-Hispanic White populations or individuals of European ancestry.^6,26,27,36,37^ Given that the incidence of pediatric glial and neuronal tumors was also lower in Hong Kong than in Asians/Pacific Islanders in the United States, environmental influence should be investigated more as a risk or protective factor in addition to genetic susceptibility.

Survival rates of patients were comparable in most histologic groups among the cohorts, including embryonal tumors, ependymomas, and GCTs. This suggests a satisfactory standard of interdisciplinary care for children with CNS tumors in Hong Kong. However, the survival for children with glial and neuronal tumors in Hong Kong was inferior, particularly for those with high-grade tumors. These worse outcomes could be due to less aggressive surgical resection in the Hong Kong cohort (Appendix Table A3) and a difference in the use of adjuvant radiotherapy. The heterogeneity within glial and neuronal tumors and the missing histologic grading in patients in the SEER cohort, despite a history of surgical resection, further complicate the interpretation of outcomes in this category.

Our study has several limitations. First, it was based on data from Hong Kong rather than the entire Chinese population. Nevertheless, before a national pediatric cancer registry becomes available in the country, the use of comprehensive population-based data from Hong Kong, where 97% of patients are of Chinese ethnicity, can be considered a reasonable surrogate. The extent of under-ascertainment in the Hong Kong cohort also was minimized by the availability of universal access to government-subsidized medical services as well as delivery of pediatric cancer treatment in designated public hospitals as members of the HKPHOSG. Second, SEER represented only 34.6% of the US population and had suboptimal ancestry designation within the Asian/Pacific Islander group, which did not allow meaningful comparison with Chinese patients only within the data set. Despite this, we based our comparison on SEER statistics rather than on the more comprehensive Central Brain Tumor Registry of the United States because of readily available follow-up data and diagnostic coding in accordance with the ICD-O-3.^4,20^ Nevertheless, future studies focusing on disease incidence may consider comparing data from the Central Brain Tumor Registry of the United States for capturing actual population data in the United States. Third, lack of data on disease progression or recurrence in both the HKPHOSG and SEER databases, as well as on details of treatment in the SEER registries, precluded the analysis on patients’ progression-free survival and comparison of outcomes by treatment received. In particular, annotation of chemotherapy and radiotherapy use in SEER did not allow us to distinguish between patients who did not receive therapy and those for whom data were missing. Fourth, diagnoses acquired from the Hong Kong and SEER registries represented the combined expertise of various radiologists and neuropathologists. The availability of central review could eliminate possible interobserver variability, whereas the next generation of tumor classification that incorporates molecular driver events is expected to facilitate the objective assignment of disease entities.^23^

In conclusion, comparison of the epidemiology of CNS tumors in Hong Kong Chinese and US children by using population-based data revealed significant differences in disease incidences, including lower rates of all brain tumors and glial and neuronal tumors and higher rates of GCTs in Chinese than in US children. These ethnic variations need to be assessed more in future studies.

## Figures and Tables

**TABLE A1:**
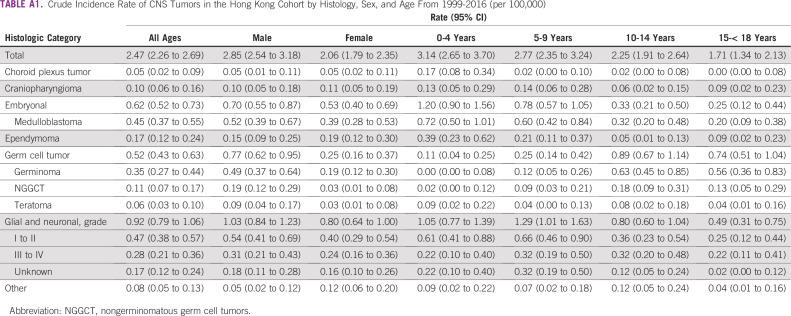
Crude Incidence Rate of CNS Tumors in the Hong Kong Cohort by Histology, Sex, and Age From 1999-2016 (per 100,000)

**TABLE A2:**
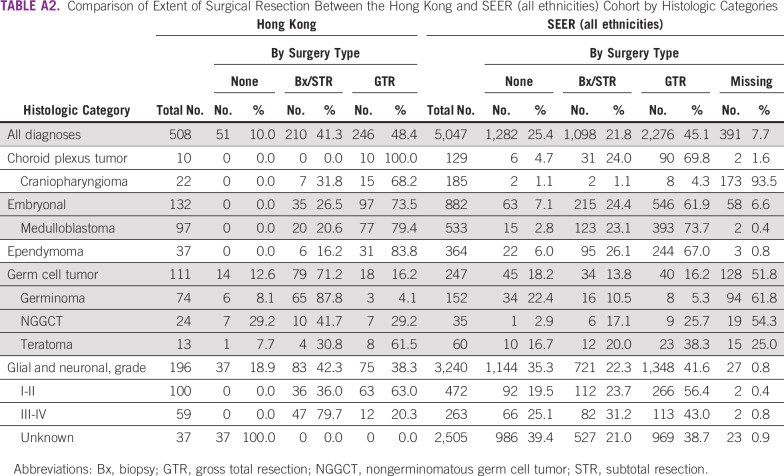
Comparison of Extent of Surgical Resection Between the Hong Kong and SEER (all ethnicities) Cohort by Histologic Categories

**TABLE A3:**
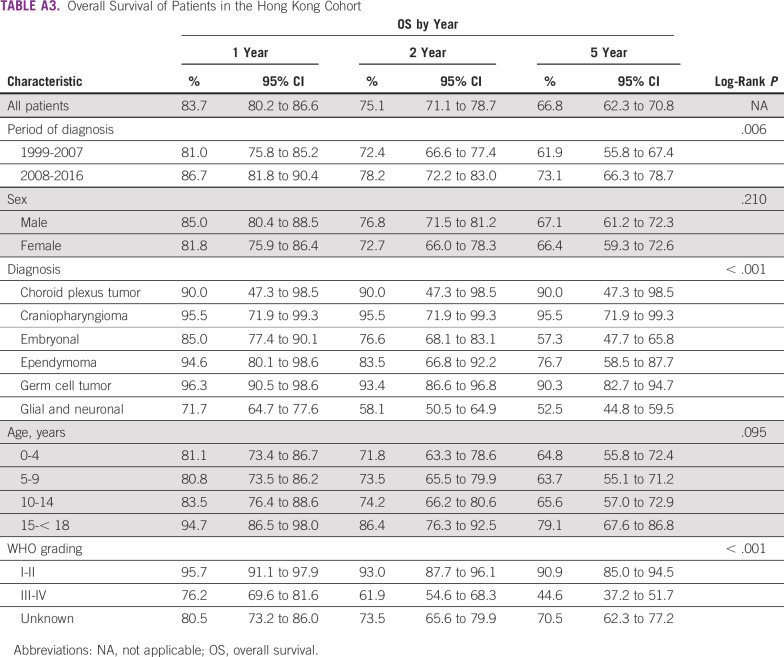
Overall Survival of Patients in the Hong Kong Cohort

**TABLE A4:**
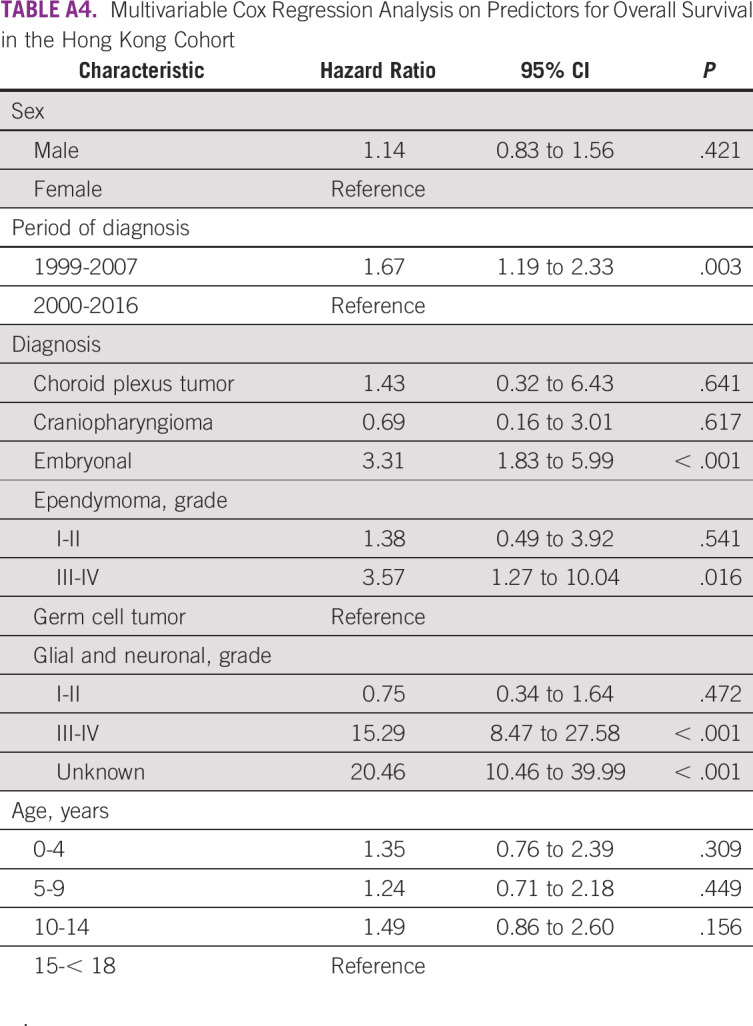
Multivariable Cox Regression Analysis on Predictors for Overall Survival in the Hong Kong Cohort

**TABLE A5:**
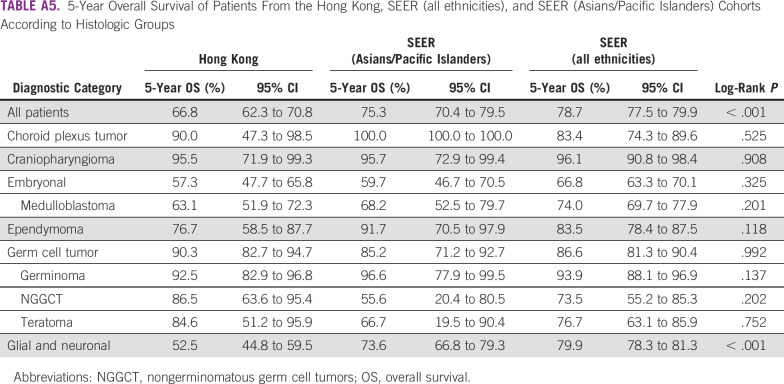
5-Year Overall Survival of Patients From the Hong Kong, SEER (all ethnicities), and SEER (Asians/Pacific Islanders) Cohorts According to Histologic Groups
